# Supplementary material for: Polyacrylamide-Based Block Copolymer Bearing Pyridine Groups Shows Unexpected Salt-Induced LCST Behavior
Source: Molecules. 2023 Mar 24;28(7):2921. doi: 10.3390/molecules28072921 (PMC10095976; doi:10.3390/molecules28072921)
Supplement: Supplementary file 1 [file molecules-28-02921-s001.zip › molecules-2271281-supplementary.pdf]

Supplementary Materials

Polyacrylamide-based Block Copolymer Bearing Pyridine Groups Shows Unexpected Salt-Induced LCST Behavior

Yunyun Tu [1,†], Dandan Fang [2,†], Wanli Zhan [2], Zengming Wei [2,*], Liming Yang [1], Pengui Shao [1], Xubiao Luo [1], Guang Yang [1,2,*]

[1] Key Laboratory of Jiangxi Province for Persistent Pollutants Control and Resources Recycle, Nanchang Hangkong University, Nanchang 330063, China

[2] Biomass Molecular Engineering Center, Anhui Agricultural University, Hefei 230036, China

*Correspondence: wzm12358@163.com (Z.W.); guangyang@ahau.edu.cn (G.Y.)

† These authors contributed equally to this work

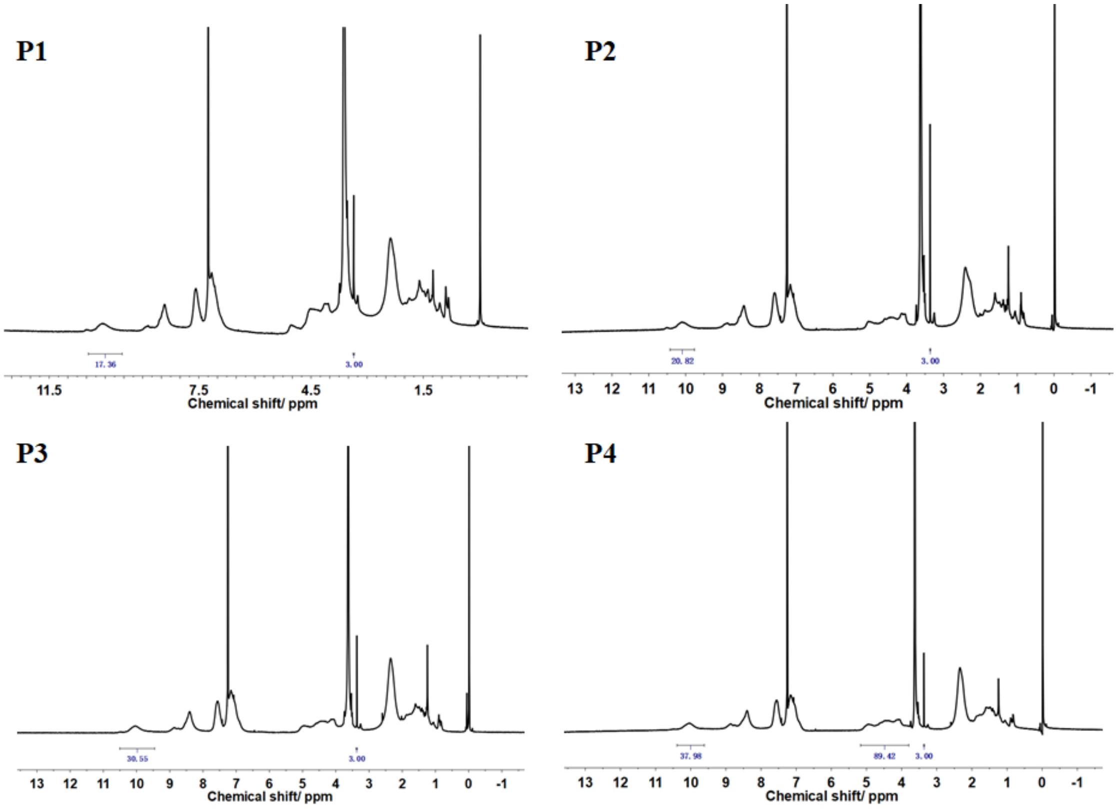

Figure S1. ^{1}H NMR spectra of P1, P2, P3 and P4 in $CDCl_3$.

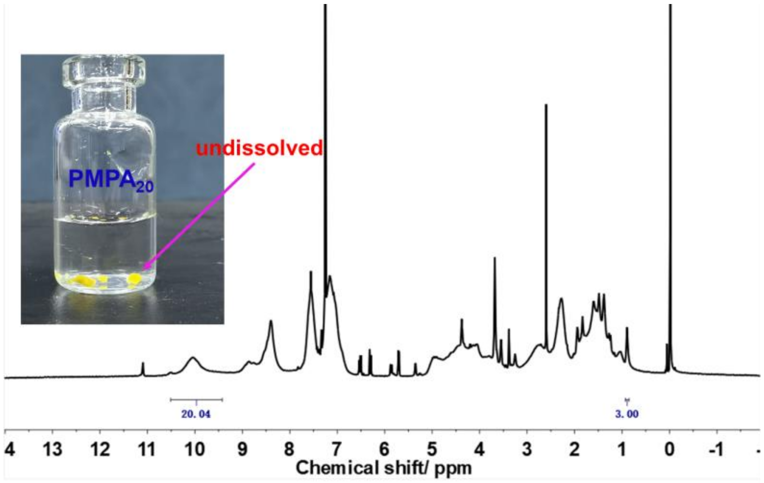

Figure S2. ^{1}H NMR spectrum of $PMPA_{20}$. The inset showed that $PMPA_{20}$ was insoluble in deionized water at room temperature.

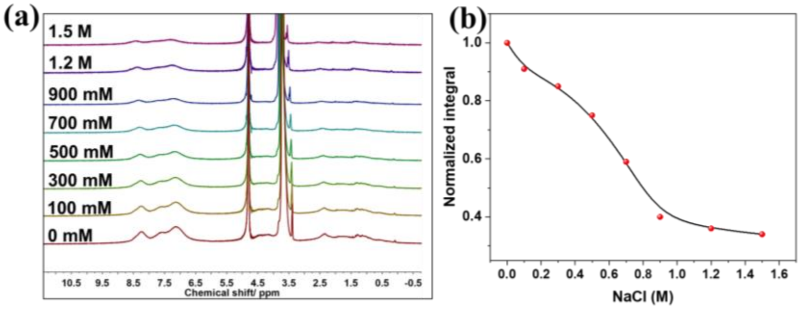

Figure S3. (a) ^{1}H NMR spectra of P2 depending on the concentration of NaCl. (b) Plots of the integration area of the protons on pyridine ring versus concentration of NaCl.

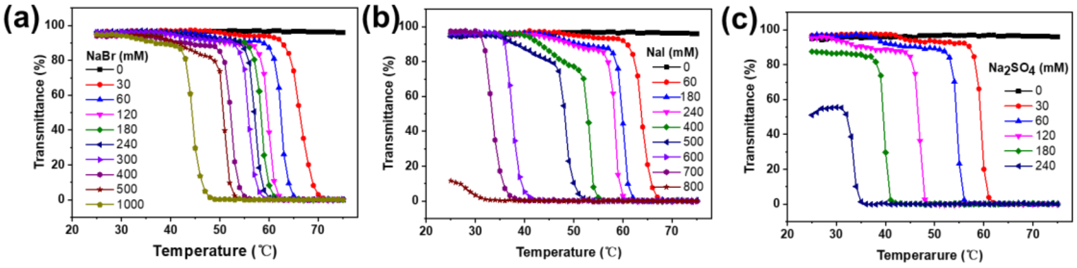

Figure S4. Effect of NaBr (a), NaI (b) and Na_2SO_4 (c) concentration on thermal response of P2.

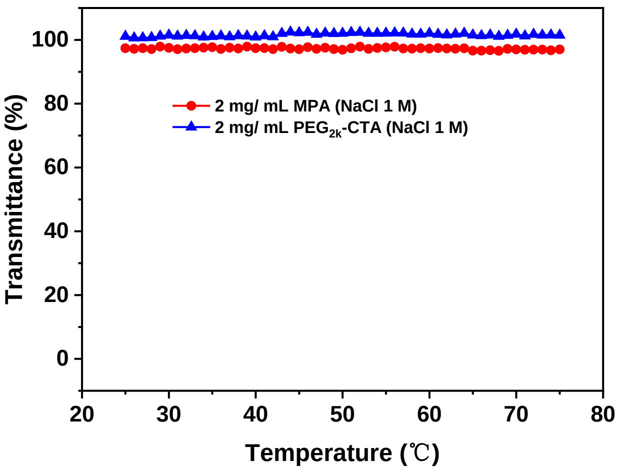

Figure S5. Turbidity change of PEG-CTA and MPA in 1 M NaCl aqueous solution.

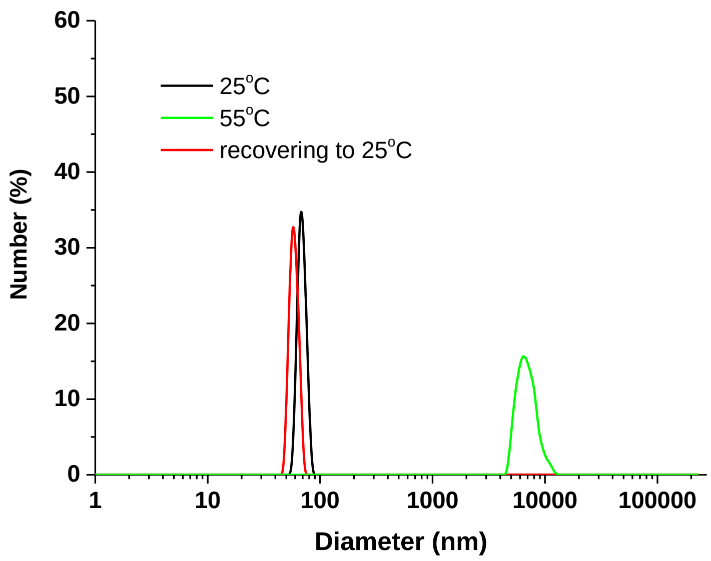

Figure S6. DLS results of P2 at 25 °C and 55 °C, respectively.

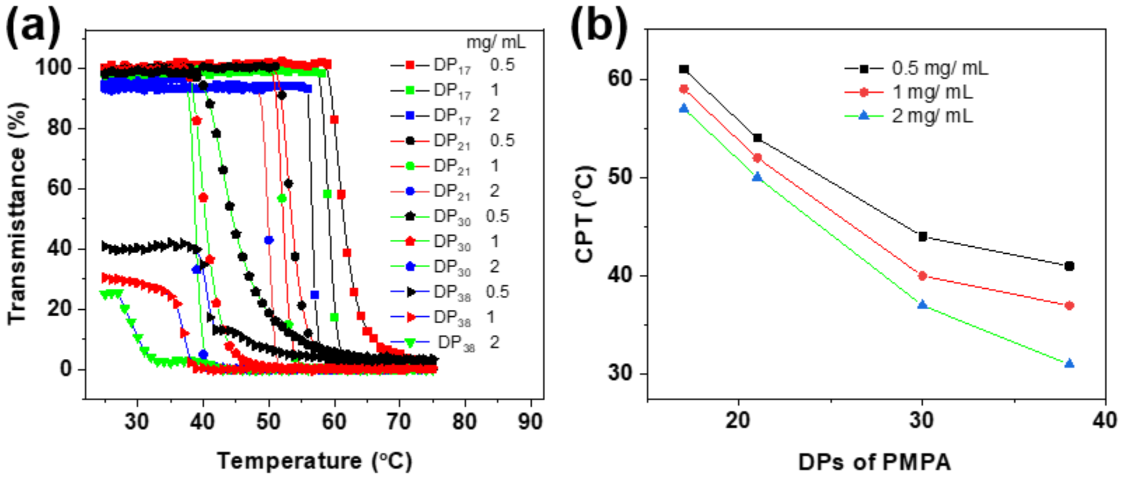

Figure S7. Effect of (a) DP of PMPA and (b) concentration on the thermal responsive behaviors of Px.
